# Patterns of Carbon-Bound Exogenous Compounds Impact Disease Pathophysiology in Lung Cancer Subtypes in Different Ways

**DOI:** 10.1021/acsnano.2c11161

**Published:** 2023-08-28

**Authors:** Jian Shen, Na Sun, Jun Wang, Philipp Zens, Thomas Kunzke, Achim Buck, Verena M. Prade, Qian Wang, Annette Feuchtinger, Ronggui Hu, Sabina Berezowska, Axel Walch

**Affiliations:** † Research Unit Analytical Pathology, Helmholtz Zentrum München − German Research Center for Environmental Health, Neuherberg 85764, Germany; ‡ Nanxishan Hospital of Guangxi Zhuang Autonomous Region, Institute of Pathology, Guilin 541002, People’s Republic of China; § Institute of Tissue Medicine and Pathology, University of Bern, Murtenstrasse 31, Bern 3008, Switzerland; ∥ Graduate School for Health Sciences, University of Bern, Mittelstrasse 43, Bern 3012, Switzerland; ⊥ Center for Excellence in Molecular Cell Science, Chinese Academy of Sciences, Shanghai 200030, People’s Republic of China; # Department of Laboratory Medicine and Pathology, Institute of Pathology, Lausanne University Hospital and University of Lausanne, Lausanne 1011, Switzerland

**Keywords:** carbon-bound exogenous compounds, carbon particles, pathophysiology, mass spectrometry imaging, non-small cell lung cancer

## Abstract

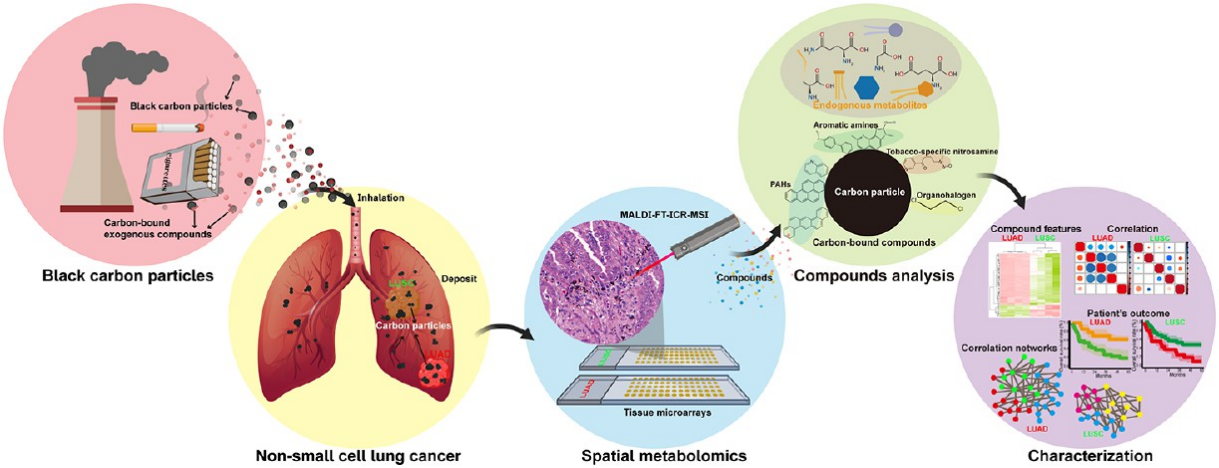

Carbon-bound exogenous compounds, such as polycyclic aromatic hydrocarbons (PAHs), tobacco-specific nitrosamines, aromatic amines, and organohalogens, are known to affect both tumor characteristics and patient outcomes in lung squamous cell carcinoma (LUSC); however, the roles of these compounds in lung adenocarcinoma (LUAD) remain unclear. We analyzed 11 carbon-bound exogenous compounds in LUAD and LUSC samples using in situ high mass-resolution matrix-assisted laser desorption/ionization Fourier-transform ion cyclotron resonance mass spectrometry imaging and performed a cluster analysis to compare the patterns of carbon-bound exogenous compounds between these two lung cancer subtypes. Correlation analyses were conducted to investigate associations among exogenous compounds, endogenous metabolites, and clinical data, including patient survival outcomes and smoking behaviors. Additionally, we examined differences in exogenous compound patterns between normal and tumor tissues. Our analyses revealed that PAHs, aromatic amines, and organohalogens were more abundant in LUAD than in LUSC, whereas the tobacco-specific nitrosamine nicotine-derived nitrosamine ketone was more abundant in LUSC. Patients with LUAD and LUSC could be separated according to carbon-bound exogenous compound patterns detected in the tumor compartment. The same compounds had differential impacts on patient outcomes, depending on the cancer subtype. Correlation and network analyses indicated substantial differences between LUAD and LUSC metabolomes, associated with substantial differences in the patterns of the carbon-bound exogenous compounds. These data suggest that the contributions of these carcinogenic compounds to cancer biology may differ according to the cancer subtypes.

## Introduction

Humans are constantly exposed to black carbon particles and carbon-bound exogenous compounds, which enter the lungs via inhalation.^1,2^ Black carbon particles are inherently toxic and serve as carriers of toxic compounds that can be released into lung tissues and metabolized over extended periods of time.^3,4^ Although foreign carbon particles are typically engulfed by resident macrophages, macrophages are incapable of efficiently degrading carbon particles.^5,6^ The engulfment of multiple carbon particles by macrophages can lead to anthracosis, a condition characterized by the visible darkening of lung tissues in concentrated areas.^7^ Because anthracosis occurs with a high prevalence, some studies have viewed anthracosis as part of a normal process, reporting that the molecules associated with accumulated carbon particles are chemically inert.^8^ However, evidence from other studies supports an association between anthracosis and lung cancer progression or carcinogenesis.^9,10^ Recently, Xie et al. analyzed gene expression and DNA methylation data from lung cells exposed to carbon nanotubes and identified four genes that are associated with the development of lung cancer.^11^ Zhang et al. found that the polycyclic aromatic hydrocarbon (PAH) accumulation in pulmonary carbon particles increases with age, which could potentially increase the risk of lung cancer development.^12^ Our previous study demonstrated that carbon-bound PAHs, tobacco-specific nitrosamines, aromatic amines, and organohalogens are strongly associated with patient outcomes, tumor development, and the tumor microenvironment in lung squamous cell carcinoma (LUSC).^13^ However, the roles of these exogenous compounds in lung adenocarcinoma (LUAD) remain unclear.

LUAD is the most common lung cancer type, accounting for 30% of all lung cancer cases and approximately 40% of all non-small cell lung cancer (NSCLC) occurrences.^14^ LUSC and LUAD, which represent the two major NSCLC histological subtypes,^15,16^ are characterized by different biological pathways and biomarkers,^17−19^ and may also exhibit differences in patterns of carbon-bound exogenous compounds or metabolomes. Recent advancements highlighting the clinical and molecular dissimilarities between LUAD and LUSC, which present distinct pathophysiologies and clinical behaviors, have led some researchers to suggest that these diseases should be treated as different cancer types rather than being classified as NSCLC subtypes.^20^ For example, current understanding of tumorigenesis indicates that LUAD develops from cells producing surfactant components, whereas LUSC arises from cells lining the airways in the lungs.^21^ LUAD is the most common type of lung cancer occurring in nonsmokers, whereas LUSC is strongly associated with smoking.^22^ LUAD and LUSC also exhibit differential gene expression patterns, with significant variations observed in the regulatory networks controlling cell proliferation, DNA replication, DNA repair, and RNA splicing.^23−26^ Mutations in receptor tyrosine kinases are frequently detected in LUAD but are rare in LUSC.^27^ The mutated tumor suppressor gene TP53 is frequently detected in early stage LUSC but only detected in late-stage LUAD.^28^ Comprehensive investigations defining the distinct molecular characteristics and metabolomes of these two major subtypes of lung cancer and the differential impacts of carbon-bound exogenous compounds on lung cancer pathophysiology remain necessary to improve diagnostic and therapeutic intervention strategies.

The highly complex and heterogeneous chemical composition of carbon particles and carbon-bound exogenous compounds in human anthracosis within the natural histological context of lung tissues has been largely unexplored and methodologically challenging. High mass-resolution mass spectrometry imaging (MSI) is capable of detecting and visualizing the discrete spatial distributions of exogenous compounds and their related metabolites in tissue sections.^29−32^ One advantage of MSI is the ability to directly overlay mass spectrometric analyses on tissue sections, allowing for correlations between molecular and histologic information.^33^ High-resolution imaging combined with molecular specificity facilitates the identification of exogenous compound characteristics at the single-pixel level. Thus, MSI can provide insights into the effects and interactions of carbon particles, exogenous compounds, and endogenous metabolites within natural cellular and extracellular contexts in human lung tissue.

In this work, we compared the carbon-bound exogenous compound patterns in LUAD and LUSC samples to improve our understanding of the differential impacts of these compounds on lung cancer pathophysiology. We used high mass-resolution matrix-assisted laser desorption/ionization (MALDI) Fourier-transform (FT) ion cyclotron resonance (ICR) mass spectrometry imaging (MALDI-FT-ICR-MSI) to analyze 11 carbon-bound exogenous compounds in LUSC and LUAD samples, revealing substantial differences in the carbon-bound exogenous compound patterns associated with these two lung cancer subtypes. PAHs, aromatic amines, and organohalogens were more abundant in LUAD than in LUSC, whereas tobacco-specific nitrosamine nicotine-derived nitrosamine ketone (NNK) was more abundant in LUSC than in LUAD. Patients with LUAD and LUSC could be clearly separated according to carbon-bound exogenous compound patterns identified in the tumor compartment. The same compounds had differential impacts on patient outcomes depending on the cancer subtype, indicating that these compounds are associated with differential contributions to LUAD and LUSC pathophysiology. Although several studies have investigated the effects of carbon particles and carbon-based materials on lung cancer development and progression, none have specifically explored the interactions between carbon-bound exogenous compounds and their metabolites or their pathophysiological contributions to different tumor types. Therefore, this study may provide a kind of insight into the impacts of carbon particles, exogenous compounds, and endogenous metabolites in human lung cancer tissue.

## Results and Discussion

### LUAD and LUSC Have Different Patterns of Carbon-Bound Exogenous Compounds

**Scheme 1:**
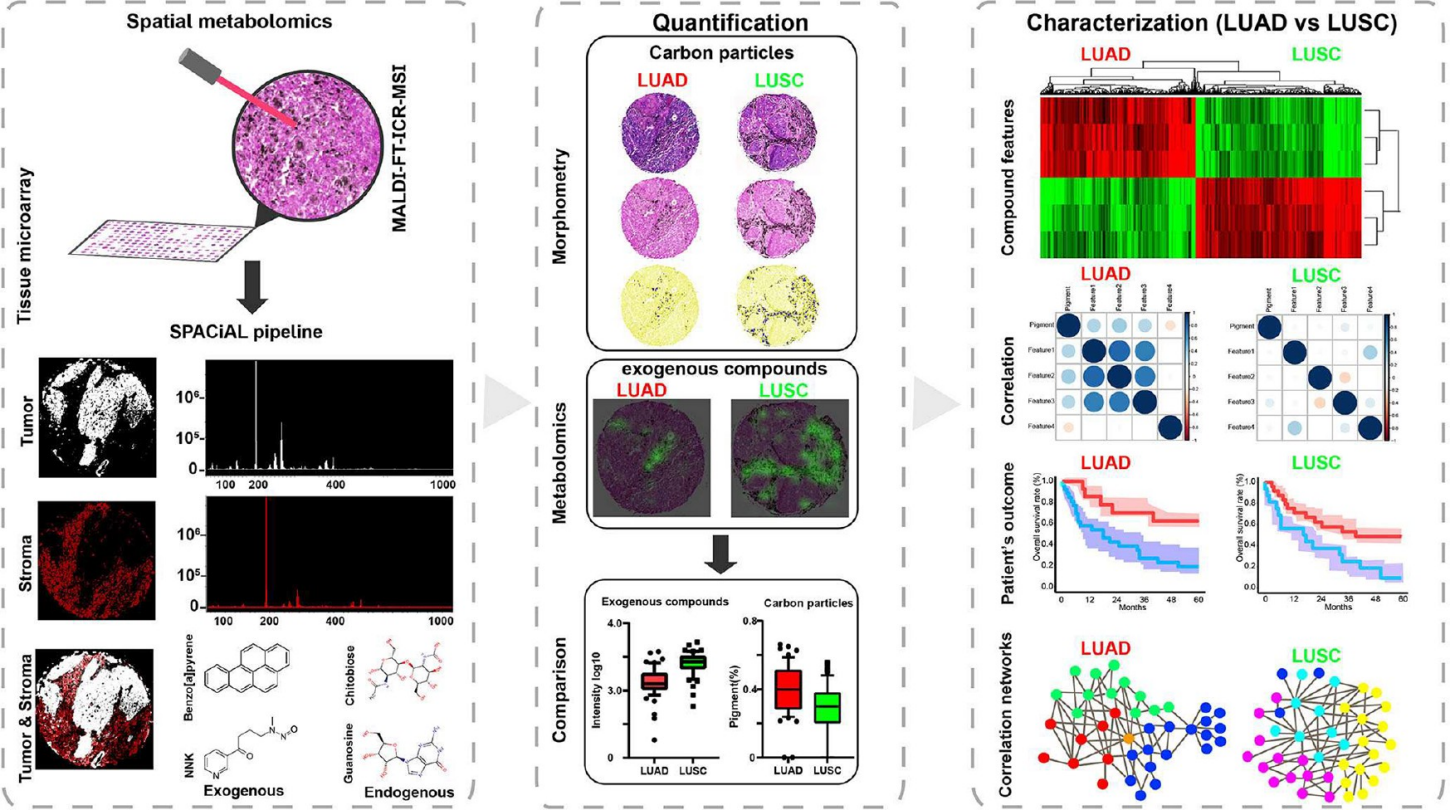
High Mass-Resolution MALDI-FT-ICR-MSI Workflow Spatial metabolomics (left): The spatial distributions of compounds were estimated by MALDI-FT-ICR-MSI, and tumor and stromal cells were automatically annotated on multiplex immunofluorescent images. Quantification (middle): The numbers of carbon particles in the LUAD (*n* = 59) and LUSC (*n* = 54) samples were quantified by morphology-based imaging analysis. Characterization (right): Carbon-bound exogenous compounds were detected by metabolomic analysis and used for hierarchical clustering and statistical analysis. LUAD, lung adenocarcinoma; LUSC, lung squamous cell carcinoma; MALDI-FT-ICR-MSI, matrix-assisted laser desorption/ionization Fourier-transform ion cyclotron resonance mass spectrometry imaging; and SPACiAL, spatial correlation image analysis.

Using the approach illustrated in Scheme 1, we examined the tumor and stromal compartments in a cohort of 113 patients with either LUAD (*n* = 59) or LUSC (*n* = 54) using high mass-resolution MALDI-FT-ICR-MSI to analyze 11 carbon-bound exogenous compounds: benzo[*a*]pyrene; dibenz[*a*,*h*]anthracene; dibenzo[*a*,*l*]pyrene; benzo[*b*]pyridine; 7-Hydroxymethyl-12-methylbenz[*a*]anthracene sulfate; NNK; 4-(methylnitrosamino)-1-(3-pyridyl)-1-butanol (NNAL); NNAL-*N*-glucuronide; *N*-hydroxy-4-aminobiphenyl; *N*-hydroxy-MeIQx; and 1,2-dichloroethane. We also compared the patterns of carbon-bound exogenous compounds in the epithelial compartments of tumors and normal tissues.

**Figure 1:**
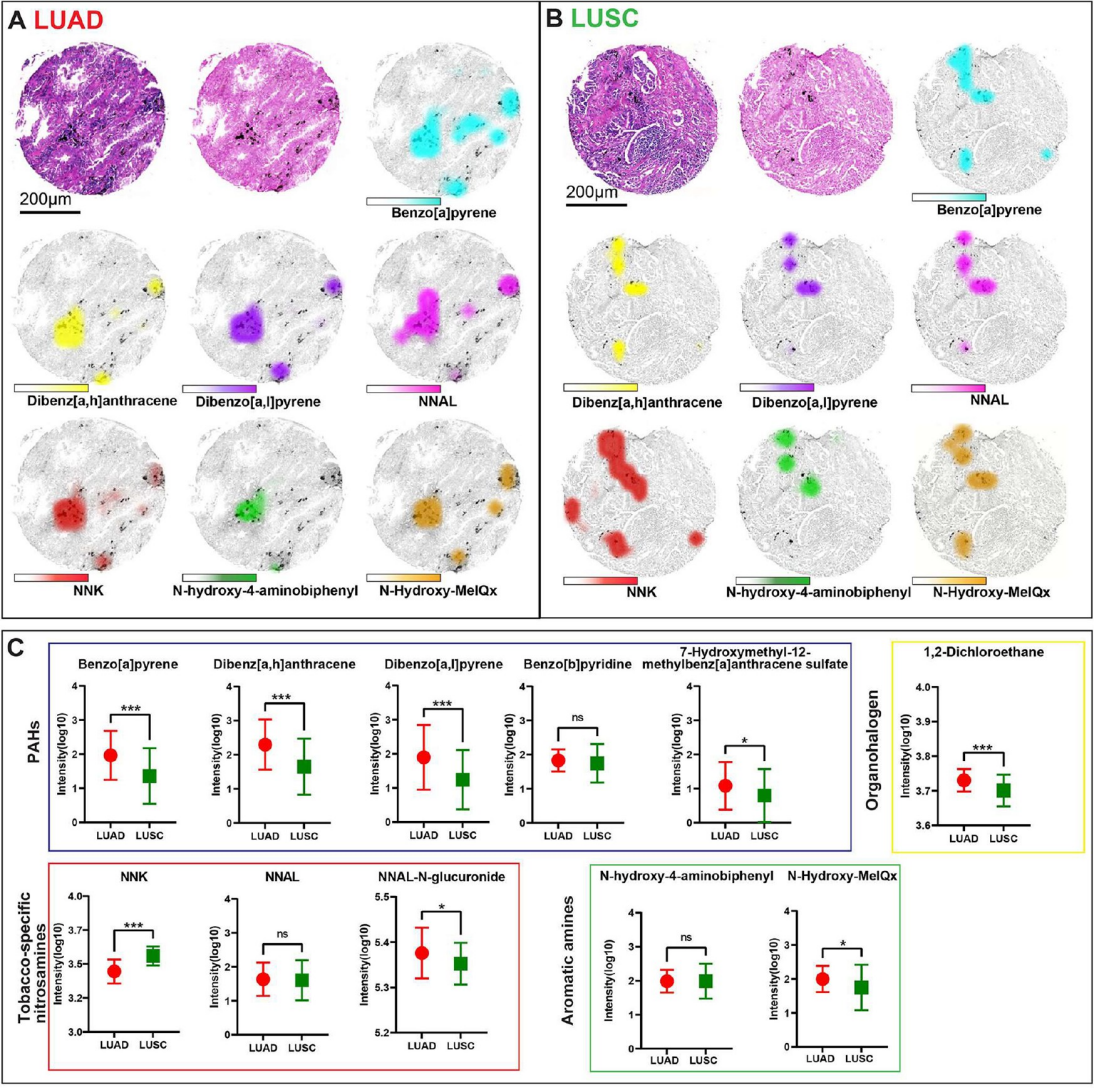
Differing patterns of carbon-bound compounds in the tumor compartments of LUAD (*n* = 59) and LUSC (*n* = 54) samples. LUAD (A) and LUSC (B) tumor tissues featuring high carbon-particle contents (top left: hematoxylin and eosin staining; top center: nuclear red staining). Ion distributions are shown for benzo[*a*]pyrene (top right), dibenz[*a*,*h*]anthracene, dibenzo[*a*,*l*]pyrene, NNAL, NNK, *N*-hydroxy-4-aminobiphenyl, and *N*-hydroxy-MeIQx. (C) Comparison of exogenous compound patterns between LUAD and LUSC samples. *p < 0.05, **p < 0.01, ***p < 0.001, ns, not significant. LUAD, lung adenocarcinoma; LUSC, lung squamous cell carcinoma; NNAL, 4-(methylnitrosamino)-1-(3-pyridyl)-1-butanol; NNK, nicotine-derived nitrosamine ketone.

In the tumor compartment, most of the exogenous compounds were more abundant in LUAD samples than in LUSC samples, including the PAHs benzo[*a*]pyrene (*p* < 0.001), dibenz[*a*,*h*]anthracene (*p* < 0.001), dibenzo[*a*,*l*]pyrene (*p* < 0.001), and 7-OH-12-methylbenz[*a*]anthracene sulfate (*p* = 0.041); the tobacco-specific nitrosamine NNAL-*N*-glucuronide (*p* = 0.017); the aromatic amine *N*-hydroxy-MeIQx (*p* = 0.016); and the organohalogen dichloroethane (*p* < 0.001); however, the tobacco-specific nitrosamine NNK (*p* < 0.001) was more abundant in LUSC samples than LUAD samples (Figure 1). In the stromal compartment, benzo[*a*]pyrene (*p* = 0.010), dibenz[*a*,*h*]anthracene (*p* = 0.002), dibenzo[*a*,*l*]pyrene (*p* = 0.016), and dichloroethane (*p* < 0.001) were more abundant in LUAD samples than in LUSC samples, whereas 7-OH-12-methylbenz[*a*]anthracene sulfate (*p* < 0.001) and NNAL-*N*-glucuronide (*p* = 0.004) were more abundant in LUSC samples than in LUAD samples (Figure S1).

**Figure 2:**
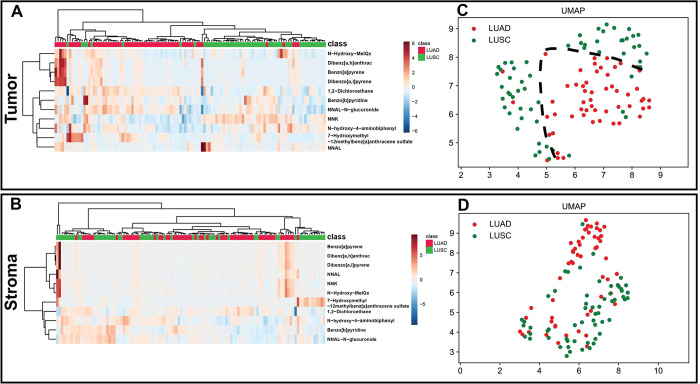
Hierarchical clustering and UMAP analysis of carbon-bound exogenous compounds in the tumor and stromal compartments of patients with LUAD (*n* = 59) and LUSC (*n* = 54). (A, B) Hierarchical clustering was performed using Metaboanalyst 4.0. The horizontal axis represents all of the samples analyzed in the study. The vertical axis denotes the 11 exogenous compounds. LUAD (red) and LUSC (green) samples are indicated on the top of the heatmap. Dendrograms for the samples are shown on the top of the heatmaps. Dendrograms for the compounds are shown on the left of the heatmaps. A blue-to-red color gradient denotes lower to higher abundance. (C, D) UMAP of LUAD and LUSC samples clustered according to the exogenous compounds detected in the tumor and stromal compartments. LUAD, lung adenocarcinoma; LUSC, lung squamous cell carcinoma; UMAP, uniform manifold approximation and projection.

Hierarchical clustering and uniform manifold approximation and projection (UMAP) analyses were able to separate the two histological tumor subtypes based on exogenous compound abundances within the tumor compartment, but no separation could be defined based on compound abundances in the stromal compartment (Figure 2). Differences in exogenous compound patterns between the tumor and normal lung tissues are shown in Figure S2. In the patients with LUAD, 7-OH-12-methylbenz[*a*]anthracene sulfate, NNAL, and *N*-hydroxy-MeIQx were more abundant in the tumor compartment than in normal lung tissue (Figure S2A). In patients with LUSC, benzo[*a*]pyridine, dibenzo[*a*,*l*]pyrene, 7-OH-12-methylbenz[*a*]anthracene sulfate, NNAL, and *N*-hydroxy-MeIQx were more abundant in the tumor compartment than in normal lung tissue (Figure S2B).

Previous studies have identified differences in the metabolic characteristics of LUAD and LUSC, but potential differences in exogenous compounds between these two NSCLC subtypes have not yet been explored.^34^ Most of the carbon-bound exogenous compounds that we examined, including PAHs, aromatic amines, and organohalogens, were more abundant in the LUAD samples than in the LUSC samples. By contrast, the tobacco-specific compound NNK was more abundant in LUSC samples than in LUAD samples, and patients with LUSC were associated with a higher smoking prevalence than those of patients with LUAD. Differences in patterns and effects of exogenous compounds between LUAD and LUSC are likely attributable to diverse factors, including cell types, physicochemical compound properties, and smoking behaviors. Different cell types in central and peripheral airways may have differential effects on exogenous compound metabolism, which might contribute to the variations in compound abundances observed between LUAD and LUSC samples.^35^ Previous studies have shown that LUSC and LUAD arise from the central and peripheral airways, respectively, and regional airway differences can influence the dose of nitrosamines in the vapor phase of tobacco smoke.^36^

Physicochemical properties, such as the octanol–water partition coefficient, water solubility, p*K*_a_, and volatility, are crucial for determining the cytotoxicity of both exogenous compounds and carbon particles and how they interact with tissue environments, including water and lipid solubility, tissue absorption, and the crossing of biological barriers (e.g., alveolar membranes).^37−42^ The physicochemical properties of the exogenous compounds assessed in this study are summarized in Table 1 (Pubchem: https://pubchem.ncbi.nlm.nih.gov/;^43^ HMDB: http://www.hmdb.ca/(44)).

**Table 1:** Physicochemical Properties of the 11 Exogenous Compounds^a^

name	PubChem CID	class	chemical formula	octanol–water partition coefficient	water solubility (g/L)	p*K*_a_	volatility
Benzo[*a*]pyrene	2336	PAHs	C_20_H_12_	6.13	1.62 × 10^–6^	15.00	SVOC
Dibenz[*a*,*h*]anthracene	5889	PAHs	C_22_H_14_	6.50	2.49	3.24	SVOC
Dibenzo[*a*,*l*]pyrene	9119	PAHs	C_24_H_14_	7.71	3.62 × 10^–6^	10.41	SVOC
Benzo[*b*]pyridine	7047	PAHs	C_9_H_7_N	2.03	6.11	4.90	VOC
7-HMBAS	54611	PAHs	C_20_H_16_O_4_S	1.75	2.20 × 10^–4^	–1.11	SVOC
NNK	47289	Nitrosamines	C_10_H_13_N_3_O_2_	0.33	1.03	3.79	SVOC
NNAL	104856	Nitrosamines	C_10_H_15_N_3_O_2_	0.48	2.64	4.79	SVOC
NNAL-*N*-glucuronide	53297450	Nitrosamines	C_16_H_23_N_3_O_8_	–1.40	0.83	3.51	SVOC
*N*-hydroxy-4-aminobiphenyl	81261	Aromatic amines	C_12_H_11_NO	2.78	0.09	5.05	SVOC
*N*-Hydroxy-MeIQx	115104	Aromatic amines	C_11_H_11_N_5_O	1.70	20.80	10.91	SVOC
1,2-Dichloroethane	11	Organohalogen	C_2_H_4_Cl_2_	1.48	8.60	1.45	VOC

a 7-HMBAS, 7-Hydroxymethyl-12-methylbenz[*a*]anthracene sulfate; NNK, 4-(Methylnitrosamino)-1-(3-pyridyl)-1-butanone; NNAL, 4-(Methylnitrosamino)-1-(3-pyridyl)-1-butanol; PAHs, polycyclic aromatic hydrocarbons; SVOC, semivolatile organic compounds; VOC, volatile organic compounds.

For example, a compound with a high octanol/water partition coefficient is more likely to accumulate in lipid-rich cells of the lung, such as alveolar type II cells and macrophages.^45^ p*K*_a_ is a measure of the acidity or basicity of a compound, such that a lower p*K*_a_ generally indicates a stronger acid, whereas a higher p*K*_a_ indicates a weaker acid. The p*K*_a_ value of a compound can also alter its charge, affecting tissue distribution by altering the ability to cross biological membranes.^46,47^ Volatile compounds can be classified according to their boiling points as very volatile organic compounds (VVOCs; boiling points ranging from 0 °C to 50–100 °C), volatile organic compounds (VOCs; boiling points ranging from 50–100 °C to 240–260 °C) or semivolatile organic compounds (SVOCs; boiling points ranging from 240–260 °C to 380–400 °C).^48^ The levels of some VOCs, such as benzaldehyde, are higher in LUAD than in LUSC samples.^49^ In our study, we found that the VOC benzo[*b*]pyridine was more abundant in the LUAD samples than in the LUSC samples. PAHs with high octanol–water partition coefficients present with slower lung clearance rates in rats^50^ and may be associated with similarly slow clearance rates in humans because they are more likely to accumulate in the lipid-rich environment of lung tissue.^51^ Some PAHs, such as benzo[*a*]pyrene, have low solubility and penetrate tissue membranes via hydrophobic attraction. After entering the body, these compounds can be deposited in lung tissue, leading to harmful effects.^52^ Because the physicochemical properties of exogenous compounds can be used to predict their behaviors in biological systems and provide insights into their likely distributions in tissues, including lung tissues, these properties should be considered when evaluating the health risks associated with compound exposure and developing strategies for detection, monitoring, and elimination.

Although both smoking and air pollution increase the risks of developing LUSC and LUAD, LUAD occurs more frequently in nonsmokers than in smokers and is strongly associated with exposure to air pollution, whereas LUSC is associated with smoking. Tobacco smoke and air pollution primarily consist of fine particles from various sources, such as insufficient biomass combustion.^53,54^ Although tobacco smoke and air pollution share some common features that contribute to lung disease, notable differences exist in the types of carcinogens that they contain. Smoking tobacco releases more than 5,200 compounds, including carcinogenic tobacco-specific nitrosamines, such as NNK, and PAHs, such as benzo[*a*]pyrene, which are important contributors to the development of cancer and other smoking-associated health problems.^55−57^ Compounds known to contribute to lung cancer in air pollution include PAHs, such as benzo[*a*]pyrene.^58^ In our study, we found a higher abundance of the tobacco-specific nitrosamine NNK in LUSC samples than in LUAD samples, whereas PAHs, such as benzo[*a*]pyrene, were more abundant in LUAD samples than in LUSC samples, suggesting that the development of LUSC and LUAD may involve different mechanisms.

The differences in carbon-bound exogenous compound patterns and their effects in LUAD and LUSC can be attributed to various factors that impact the metabolism, accumulation, and distribution of these compounds, including distinct transcriptomic profiles, alterations in protein expression, or genetic driver mutations. At the transcriptomic level, previous studies identified cytochrome P450 family 1 subfamily a member 1 (CYP1A1) as being involved in the metabolism of exogenous compounds, including PAHs such as benzo[*a*]pyrene.^59^ CYP1A1 expression can be detected in LUAD but not in most LUSC.^60^ At the protein level, epidermal growth factor receptor (EGFR) expression is more frequently detected in LUAD samples than in LUSC samples,^61,62^ and EGFR expression may increase carbon particles uptake.^63,64^ At the genetic level, recent studies have identified several single-nucleotide polymorphisms in genes involved in the metabolism of both endogenous and exogenous compounds that are associated with increased risks for lung cancers development, especially LUAD, in individuals who have never smoked.^65^

### The Same Carbon-Bound Exogenous Compounds Have Differential Effects on Patient Outcomes in LUAD and LUSC

**Figure 3:**
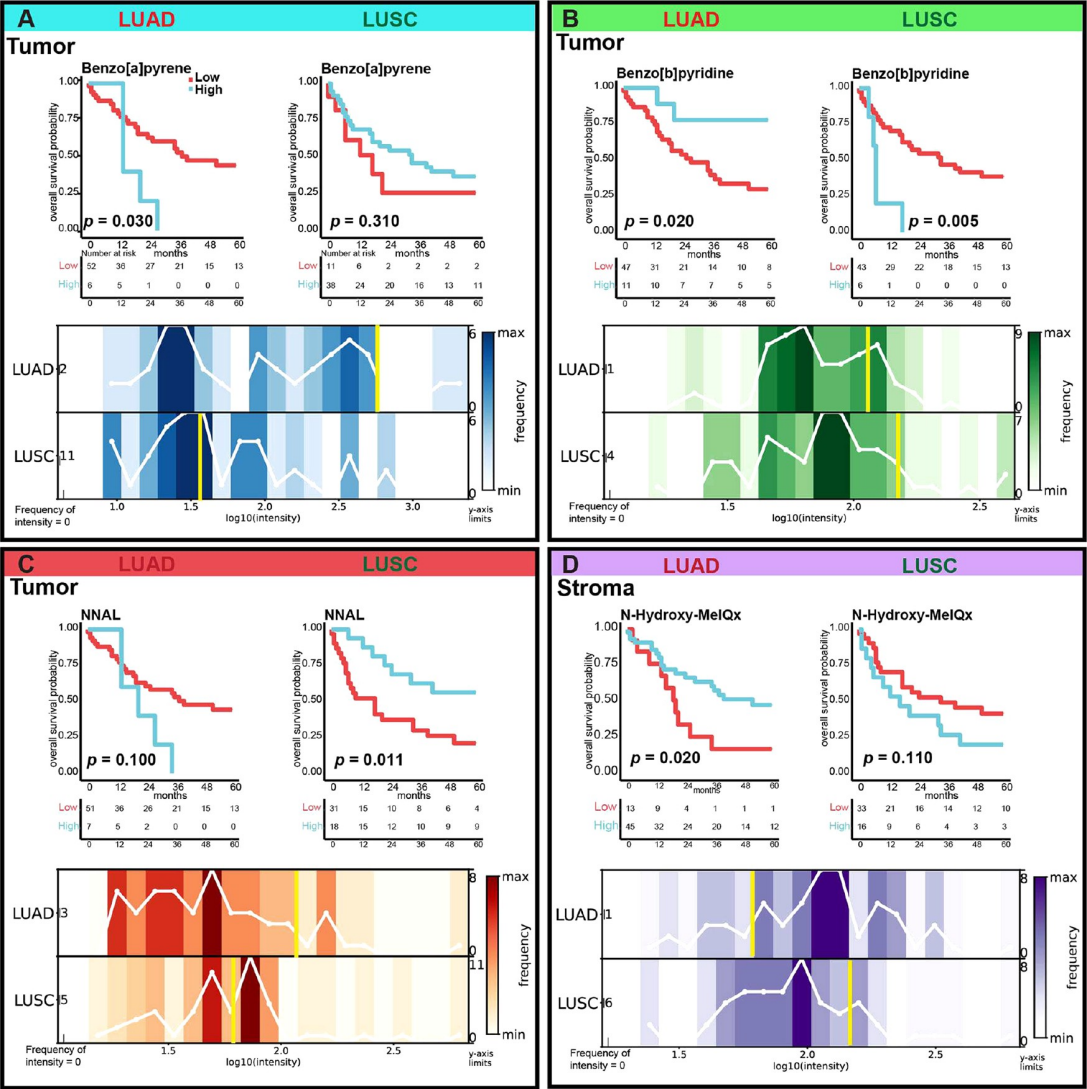
Kaplan–Meier survival analyses (top) and distributions of exogenous compound abundances (bottom). Kaplan–Meier survival analyses revealing that low tumoral benzo[*a*]pyrene (A) levels were associated with better prognosis in LUAD (*n* = 58) but trended toward a worse prognosis in LUSC (*n* = 49). High levels of tumoral benzo[*b*]pyridine (B) were associated with better prognosis in LUAD but worse prognosis in LUSC. High NNAL (C) levels were associated with a better prognosis in LUSC but trended toward a worse prognosis in LUAD. High stromal *N*-hydroxy-MeIQx (D) levels were associated with better prognosis in LUAD but trended toward a worse prognosis in LUSC. Bottom graphs are noncumulative histograms showing the distributions of compound abundances, including the intensity thresholds used to classify samples as having high or low compound levels (yellow). The counts of intensity = 0 are displayed on the left side of the *y*-axis. On the right side of the*y*-axis, the maximum number of patients in a bin is shown. LUAD, lung adenocarcinoma; LUSC, lung squamous cell carcinoma; NNAL, 4-(methylnitrosamino)-1-(3-pyridyl)-1-butanol.

**Figure 4:**
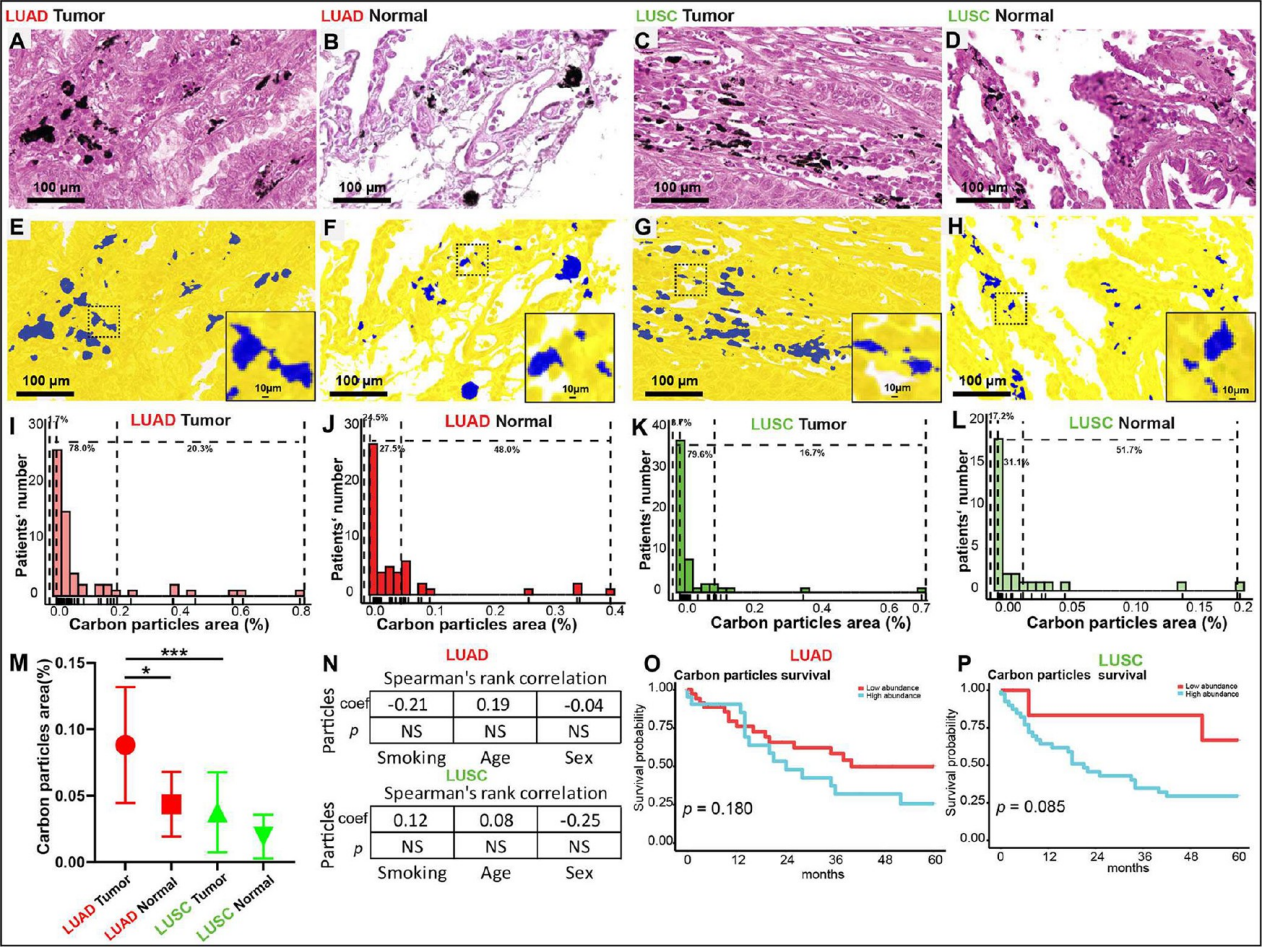
Anthracosis in normal lung and NSCLC tissue. (A–D) Tumor and normal tissues were stained with hematoxylin and eosin and counterstained with nuclear red, revealing anthracosis deposits. (E–H) Segmentation (blue) by image analysis for anthracosis quantification. (I, J) The quantities and prevalence of carbon particles in clusters were evaluated in both tumor and normal tissues from patients with LUAD (*n* = 59). (K, L) The quantities and prevalence of carbon particles in clusters were assessed in both tumor and normal tissues from patients with LUSC (*n* = 54). The dashlines in the histogram indicate the percentage of patients divided into three categories: no, low, or high pigment content. (M) Comparison of the numbers of carbon particles in clusters in tissues from patients with LUAD and LUSC. (N) Spearman’s rank correlation showing the associations between the total area of carbon particles in clusters and various characteristics. (O, P) Kaplan–Meier survival analysis showing that carbon particle abundance in clusters is not correlated with patient survival. *p < 0.05, ***p < 0.001, NS, not significant. NSCLC, non-small cell lung cancer; LUAD, lung adenocarcinoma; LUSC, lung squamous cell carcinoma.

We compared the prognostic associations of carbon-bound exogenous compounds with LUAD and LUSC outcomes and found that the same carbon-bound exogenous compounds had differential effects on patient outcomes depending on the lung cancer subtype (Figure 3). In the tumor compartment of LUAD, high benzo[*a*]pyrene levels (*p* = 0.030) and low benzo[*b*]pyridine levels (*p* = 0.020) were both associated with poor prognosis (Figure 3A and 4B top), whereas low NNAL levels (*p* = 0.100) trended toward an association with favorable prognosis (Figure 3C top). In the tumor compartment of LUSC, high NNAL levels (*p* = 0.011) and low benzo[*b*]pyridine levels (*p* = 0.005) were each associated with a favorable prognosis (Figure 3A–C top). In the stromal compartment of LUAD, high *N*-hydroxy-MeIQx levels (*p* = 0.020) were associated with a favorable prognosis, but the opposite trend was observed for *N*-hydroxy-MelQx levels in the stromal compartment of LUSC (*p* = 0.110).

Some exogenous compounds had differential effects on patient outcomes in LUAD and LUSC, suggesting that exogenous compound patterns may drive LUAD and LUSC pathophysiology. For example, high benzo[*a*]pyrene levels were associated with poor prognosis in LUAD but were not associated with survival in LUSC. The same compounds may have differential effects on patient outcomes in LUAD and LUSC for various reasons. First, abundance–response relationship may exist between PAH dose and patient survival, and PAHs are more abundant in LUAD than in LUSC. Second, an abundance–response relationship between PAHs and DNA methylation has been reported in lung cancer.^66^ DNA methylation is tissue-specific, distinct DNA methylation patterns have been identified in LUAD and LUSC,^67,68^ and DNA methylation anomalies are associated with carcinogenesis and patient survival in lung cancer.^69,70^ Therefore, the differential effects of PAHs across different lung cancer subtypes may be mediated by differences in DNA methylation patterns and frequencies. Third, PAHs and halogenated aromatic hydrocarbons are aryl hydrocarbon receptor (AhR) ligands,^71^ and abnormal AhR expression and activity have been reported in lung cancer.^72−74^ Immunoreactivity analysis of tissues from patients with cancer revealed more prevalent AhR overexpression in LUAD tissues than in LUSC tissues.^75,76^ AhR overexpression is associated with increased tumor cell proliferation and survival in lung cancer, and AhR antagonists exhibit anticancer activity, indicating a potential role for AhR in tumor pathophysiology.^77−79^ Thus, differences in the AhR expression levels might also explain the different prognostic associations observed for the same exogenous compounds in LUAD and LUSC.

Patients with LUSC had a higher smoking rate than patients with LUAD (*p* < 0.001). Tumoral NNK levels were positively correlated with smoking in LUAD (*r* = 0.32, *p* = 0.027) but showed no significant correlation with smoking in LUSC (*r* = 0.15, *p* = 0.303); however, high NNK levels were associated with poor prognosis in both LUAD (*p* = 0.003) and LUSC (*p* = 0.020). Survival analyses based on exogenous compound levels are shown in Figure S3. NNK can induce DNA adducts at a high frequency, inhibiting DNA repair mechanisms in human cells and resulting in the accumulation of DNA damage, which could potentially contribute to the development of cancer.^80^ Additionally, NNK can interact with various signaling pathways, such as the phosphoinositide 3-kinase–protein kinase B–mechanistic target of rapamycin pathway, and transcription factors, including hypoxia-inducible factor-1α, which are involved in tumor growth, survival, and angiogenesis.^81^ Although the mechanisms underlying NNK-induced metastasis and poor prognosis are complex and are not fully understood, studies indicate that NNK can activate a signaling pathway involving c-Src–protein kinase C iota–focal adhesion kinase to promote tumor invasion and migration, contributing to lung cancer development and metastasis.^82^ Although no association exists between NNK and metastasis in either LUAD (*p* = 0.96, coefficient = −0.05; Figure S4A) or LUSC (*p* = 0.83, coefficient = −0.06; Figure S4B), other factors, such as genetic mutations or differences in the tumor microenvironment, may contribute to the observed association between NNK levels and poor prognosis in both LUAD and LUSC.

### Carbon Particles Are More Abundant in LUAD than in LUSC

We measured the abundances of carbon particles in LUAD and LUSC samples to determine whether they correlate with patient survival or tumor subtypes. We also compared carbon particle abundances between tumor and normal lung tissues to assess whether carbon particle levels correlate with tumor presence. Image analysis showed detectable carbon deposits in both tumor and normal tissues from patients with LUAD (Figure 4A,B) and those with LUSC (Figure 4C,D). Digital image analysis was used to quantify carbon particles in LUAD, LUSC, and normal tissue samples (Figure 4E–H), and the carbon particle distributions are shown in Figure 4I–L.

Carbon particles were more abundant in LUAD tumor tissues than in LUSC tumor tissues (*p* < 0.001; Figure 4M), but no significant differences were observed for normal tissues. Carbon particles were more abundant in stromal tissues from patients with LUAD than in those from patients with LUSC (*p* < 0.001; Figure S5). No significant differences in carbon particles were observed between tumor and stromal tissues from patients with LUAD (*p* = 0.244; Figure S5). However, in tissues from patients with LUSC, carbon particles were more abundant in stromal tissues than in tumor tissue (*p* = 0.021; Figure S5). Carbon particles were more abundant in tumor tissues than in normal lung tissues from patients with LUAD (*p* = 0.02), but no significant differences were observed in carbon particle abundances between tumor tissues and normal lung tissues from patients with LUSC (Figure 4M). Carbon particle abundance was not associated with clinical features, such as sex, smoking behavior, or age (Figure 4N), or with overall survival, regardless of the histologic type (Figure 4O and 4P).

In tumors, carbon particle abundance correlates with the macrophage clearance rate, as macrophages are primarily responsible for removing carbon particles from the lungs. Studies have revealed that carbon nanotubes undergo degradation within macrophages, shedding light on the biological pathways involved in the removal of carbon particles.^83^ Others found that lung macrophages degrade carbon particles through an oxidative pathway involving superoxide and peroxynitrite. This pathway has implications for the development of lung diseases, including fibrosis and carbon nanotube induced cancer. Differences in macrophage subtypes and their abundances in LUAD and LUSC may also contribute to differences in the metabolism of carbon particles and exogenous compounds.^84^ Previous studies have shown that fatty acid-binding protein 4-expressing macrophages, which are primarily detected in LUAD, are closely associated with phagocytosis and immune responses, whereas secreted phosphoprotein 1-expressing macrophages, which are primarily enriched in LUSC, exhibit angiogenesis-related gene expression.^85^ Smoking elevates the presence of macrophages in the immune microenvironment in lung cancer patients, indicating that smoking may also play a role in the observed variations in the exogenous compound metabolism between LUAD and LUSC.

Carbon particles present with distinct and size-dependent physical and chemical properties,^86^ and the deposition of carbon particles depends on their aerodynamic diameter (dp). Coarse particles (2.5 μm < dp < 10 μm) are primarily retained in the upper respiratory tract and central airways; fine particles (dp < 2.5 μm) are deposited in the tracheobronchial region; and ultrafine particles (dp < 0.1 μm) are deposited in the pulmonary and alveolar regions.^87^ Fine and ultrafine particles may penetrate deeply within the lungs and bronchioles and may reach the circulatory system, allowing systemic effects on distant organs.^88^ LUAD typically arises from the bronchial or alveolar epithelium of the peripheral airways, areas where fine and ultrafine particles are able to accumulate.^89^ By contrast, LUSC originates from the areas likely to accumulate coarse and fine particles, such as the bronchial epithelium of the central airways.^90^ High levels of carbon particle deposition have been associated with increased risks of developing NSCLC,^91,92^ and a significant correlation has been reported between fine particle levels and lung cancer incidence in EGFR-driven cases. Another study revealed comparable associations (reported as hazard ratios) between LUAD development and both coarse and fine carbon particle deposition,^93^ and a close correlation between particle size and PAH levels has also been reported, with nanosized particles (0.01 < dp < 0.056 μm) associated with the highest PAH levels, followed by ultrafine, fine, and coarse particles. In our study, higher carbon particle levels were associated with higher levels of some exogenous compounds, including benzo[*a*]pyrene, dibenz[*a*,*h*]anthracene, dibenzo[*a*,*l*]pyrene, 7-OH-12-methylbenz[*a*]anthracene sulfate, NNAL-*N*-glucuronide, *N*-hydroxy-MeIQx and dichloroethane in LUAD samples than in LUSC samples, indicating that carbon particle levels may be involved in LUAD development. However, we often found carbon particles in clusters, preventing the assessment of individual carbon particles. Based on previously reported associations between carbon particle size and particle deposition in the lungs,^94^ we speculate that the carbon particles deposited in LUAD tissues are likely to be fine and ultrafine particles, whereas coarse and fine particles are more likely to be deposited in LUSC tissues, suggesting differences in the carbon particle diameters and abundances associated with each type of lung cancer.

### Correlation Networks of Metabolites and Carbon-Bound Exogenous Compounds Reveal Substantially Different Metabolomes in LUAD and LUSC

**Figure 5:**
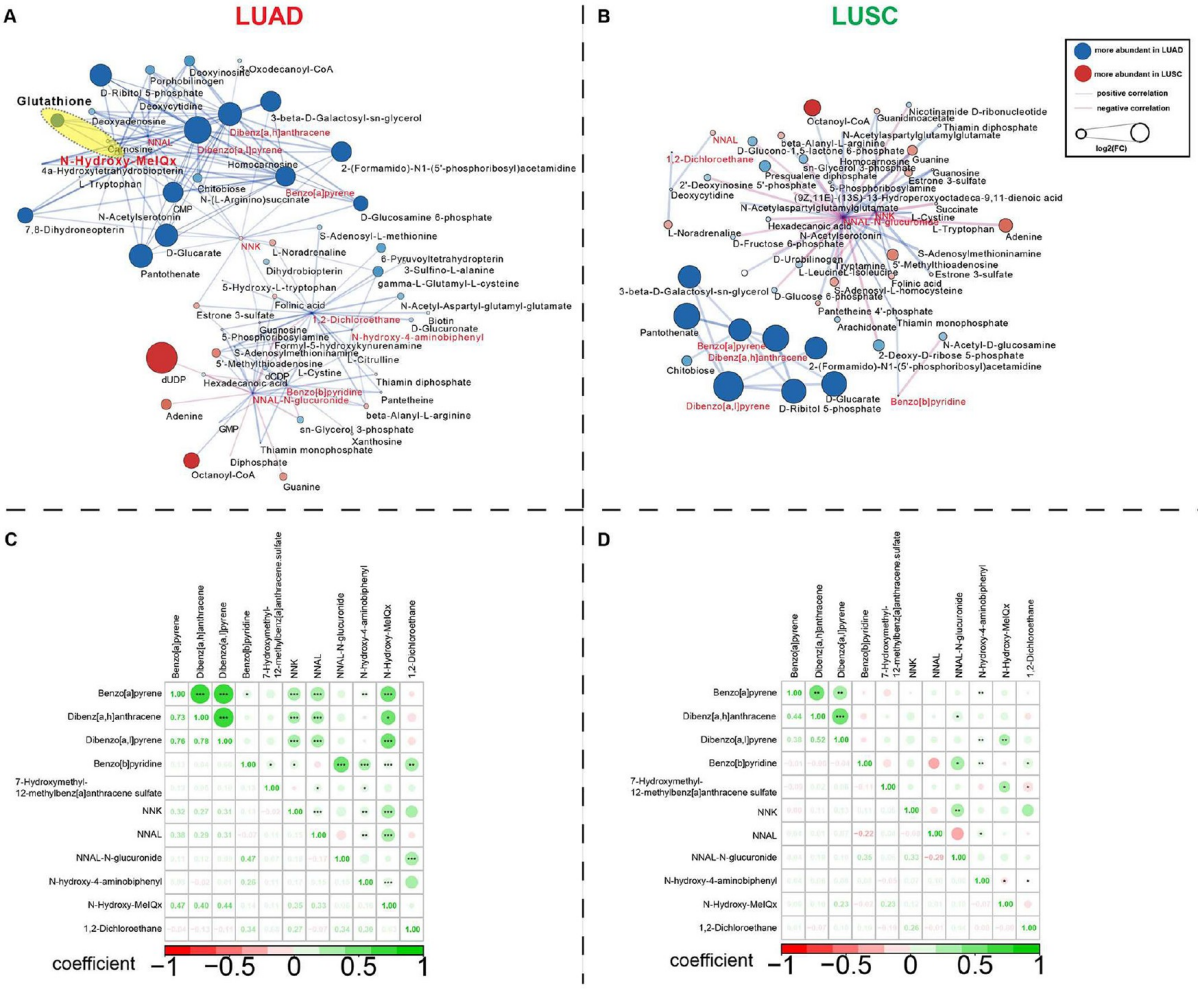
Correlations of endogenous metabolites with the exogenous compounds in LUAD (*n* = 59) and LUSC (*n* = 54). Correlation networks for LUAD (A) and LUSC (B). Correlations between exogenous compounds and endogenous metabolites were calculated and filtered (*p* < 0.01). Higher *N*-hydroxy-MeIQx levels are associated with higher glutathione level (highlighted in yellow). Node colors indicate whether individual endogenous (black) or exogenous (red) compounds were more abundant in LUAD (blue) or LUSC (orange) tissues. Node sizes correlate with the absolute log_2_ (fold-change) value. Edges represent positive (blue) and negative (pink) correlations between exogenous compounds and endogenous metabolites. (C, D) Pairwise correlations between the 11 exogenous compounds. *p < 0.05, **p < 0.01, ***p < 0.001. LUAD, lung adenocarcinoma; LUSC, lung squamous cell carcinoma; NNK, nicotine-derived nitrosamine ketone; NNAL, 4-(methylnitrosamino)-1-(3-pyridyl) 1-butanol.

We performed a network analysis to investigate metabolic tumor characteristics related to the presence of carbon-bound exogenous compounds in LUAD and LUSC tissues. Most of the endogenous metabolites identified in the LUAD network were associated with amino acid metabolism and nucleotide metabolism (Figure 5A), whereas most of the endogenous metabolites identified in the LUSC network were associated with amino sugar and nucleotide sugar metabolism, revealing substantially different metabolomes between LUAD and LUSC tissues.

In the network analysis, the metabolites with the best ability to discriminate between LUAD and LUSC were primarily nucleotides, such as deoxyuridine diphosphate (dUDP), 2-(formamido)-N1-(5′-phosphoribosyl) acetamidine, cytidine monophosphate (CMP), and adenine. In LUAD, five carbon-bound exogenous compounds were part of a dense cluster containing endogenous metabolites. In this cluster, higher levels of the purine metabolites deoxyinosine (*r* = 0.745) and deoxyadenosine (*r* = 0.665) and the pyrimidine metabolites CMP (*r* = 0.823) and deoxycytidine (*r* = 0.600) were linked to higher levels of benzo[*a*]pyrene (Figure 5A). In another dense cluster, including four exogenous compounds and endogenous metabolites, dUDP (*r* = 0.465) and guanine (*r* = 0.499) levels were negatively associated with NNAL-*N*-glucuronide levels (Figure 5A). The correlation network for LUSC differed substantially from that for LUAD (Figure 5B). For example, NNAL-*N*-glucuronide, which was detected in a dense cluster of metabolites, had the most correlations with the endogenous metabolites in the LUSC network (*r* = 0.796). The pairwise correlations between exogenous compounds were primarily positive in both LUAD (Figure 5C) and LUSC (Figure 5D).

Fundamentally different correlations between endogenous metabolites and exogenous carbon-bound compounds were identified in LUAD and LUSC. Our results suggest that the metabolite *N*-hydroxy-MeIQx is associated with nucleotide metabolism and DNA damage in LUAD. Nucleotide metabolism is enhanced in the presence of DNA damage,^95^ as increased DNA damage leads to increased DNA repair, increasing nucleotide demand.^96^ However, the functional deregulation of DNA repair is a common feature of highly aggressive human malignancies.^97^ Several studies have demonstrated differences between the gene expression profiles related to signaling pathways in LUAD and LUSC.^98^ For example, the gene encoding p53 is frequently mutated in lung tumors, but the frequency of p53 mutation differs between LUAD and LUSC. p53 mutations may result from DNA damage induced by exogenous carcinogens, such as PAHs, and PAH metabolites, such as benzo[*a*]pyrene-diol-epoxide, can reduce the speed of DNA repair.^99^ Slowly repaired DNA damage hotspots correspond to mutational hotspots observed in lung cancer.^100^

According to our network analysis, *N*-hydroxy-MeIQx levels were higher in LUAD samples than in LUSC samples and were positively correlated with glutathione levels, consistent with the findings of prior studies and leading to an increased antioxidant capacity and greater resistance against oxidative stress.^101^ Another study demonstrated that MeIQx administration increased the formation of 8-hydroxy-2′-deoxyguanosine, a marker of oxidative DNA damage, in the liver.^102^ Because glutathione is a crucial antioxidant that plays a central role in cellular detoxification and protection against oxidative damage,^103^ increased glutathione levels may mitigate oxidative damage in tumor cells in the context of *N*-hydroxy-MeIQx-induced oxidative stress. High glutathione levels are often associated with drug-resistant tumors, suggesting that glutathione may offer resistance against certain types of exogenous compounds.^104^

In LUSC tissues, NNAL-*N*-glucuronide was detected in a dense cluster of metabolites and was more highly correlated with endogenous metabolite levels than the other examined exogenous compounds. NNAL-*N*-glucuronide is a detoxification product of NNAL that may serve as an indicator of a favorable prognosis. Although NNAL levels were similar between LUAD and LUSC tissues; NNAL-glucuronide levels were higher in LUAD tissues than in LUSC tissues, suggesting that NNAL detoxification may occur at a higher rate in LUAD than in LUSC, further contributing to the observed differential effects of exogenous compounds on patient outcomes in LUAD and LUSC. Other factors, such as differences in the microenvironment or immune response, could also contribute to these differences, and the potential involvement of multiple mechanisms was highlighted by the lack of any significant correlations between NNAL-glucuronide and NNAL levels and black carbon particle deposition (Figure S6), suggesting that the black carbon particle deposition and NNAL-glucuronide formation are differentially regulated. In addition, the NNAL-glucuronide levels may not directly reflect either NNAL or carbon particle levels, as NNAL-glucuronide is a detoxification product that may be influenced by individual differences in metabolism and clearance.

## Study Limitations

Our study has several notable limitations. First, our analysis focused on only 11 carbon-bound exogenous compounds despite the likely existence of hundreds or more exogenous compounds that may contribute to the differences between LUAD and LUSC within the highly complex bioactive context of lung tissues.^105^ The 11 exogenous compounds that our present study focused on were selected, based on their detection in anthracotic lung tissues and their prior reported associations with lung cancer pathophysiology.

Second, mass spectra were acquired by high mass-resolution MALDI-MSI over a range of 75–1000 Da, and any compounds outside of this range were not detected. We used a 9-aminoacridine matrix, but matrix materials can affect the detection capacity by altering the spectrum of analytes; thus, additional compounds might be detected when using a different matrix. In addition, MALDI-MSI has a limited capacity to detect previously described anthracosis-associated toxic or carcinogenic elements, such as cadmium, arsenic. More appropriate methods, such as laser ablation-inductively coupled plasma MSI that offer superior sensitivity for the detection of toxic elements, should be considered for future studies.

Third, our approach offers a limited spatial resolution. Although our approach allows for the measurement of clusters of carbon particles, we are unable to analyze individual nanoparticles on the nanometer scale. Similar limitations exist for currently available single-cell analyses as particle clusters are often clustered within macrophages; however, single-cell metabolic analyses would be of great immunological interest. The ongoing development of MSI at single-cell resolution may facilitate such analyses in the future. Despite its limitations, MALDI-MSI remains a valuable tool for providing insights into the highly complex effects and interactions of carbon particles, carbon-bound exogenous compounds, and related endogenous metabolites in human lung cancer tissue.

## Conclusion

In summary, we showed that the two most common histologic NSCLC subtypes, LUAD and LUSC, were associated with different patterns of carbon-bound exogenous compounds with differential impacts on lung cancer pathophysiology and patient outcomes. Our findings also revealed that carbon particles were more abundant in LUAD tissues than in LUSC tissues. Understanding the differing patterns of carbon-bound exogenous compounds associated with different NSCLC subtypes is likely to provide important clues regarding the effects of exogenous compounds on tumor pathophysiology.

## Materials and Methods

### Patient Cohort

This retrospective study was conducted using matched primary resected tumor and normal lung tissue samples collected from 59 (52.2%) patients with LUAD and 54 (47.8%) patients with LUSC at the Institute of Tissue Medicine and Pathology of the University of Bern between January 2000 and December 2016, as described previously.^106^ The median age at surgery was 61 years (interquartile range [IQR] 40–82 years) for patients with LUAD and 67 years (IQR 39–84 years) for patients with LUSC. All tumors were at a locally advanced stage, defined by the presence of mediastinal lymph-node metastases (pN2 or pN3). Tissue microarrays (TMAs) were constructed from formalin-fixed and paraffin-embedded (FFPE) tissue blocks, as previously described. Briefly, 0.6 mm diameter tissue cylinders were punched from annotated regions of FFPE tissue blocks by a pathologist specializing in lung pathology (SB). FFPE samples were analyzed in parallel by high mass-resolution MALDI-FT-ICR-MSI (Scheme 1). Overall survival was defined as the time from resection to death by any cause. The clinicopathological characteristics of the patients, including smoking status, are summarized in Table 2. This study was approved by the Cantonal Ethics Commission of the Canton of Bern (KEK 2017–00830) in accordance with the Swiss Human Research Act and Declaration of Helsinki.

**Table 2:** Baseline Characteristics of Patient Cohorts^a^

	Primary resection (*N* = 113)	
	LUSC (*N* = 54)	LUAD (*N* = 59)
**Sex (male/female)**	48/6 (88.8%/11.2%)	27/32 (45.8%/54.2%)
**Age, years (median)**	39–84 (67)	40–82 (61)
**Smoking (yes/no)**	48/2 (88.8%/3.7%)	27/12 (45.8%/20.3%)
Nonsmoker	2 (3.7%)	12 (20.3%)
Active smoker	19 (35.2%)	15 (25.4%)
Ex-smoker	29 (53.7%)	12 (20.3%)
No record	4 (7.4%)	20 (34.0%)
**pT**		
pT1	6 (11.5%)	11 (18.6%)
pT2	11 (21.2%)	23 (40.1%)
pT3	17 (32.6%)	15 (25.4%)
pT4	18 (7.7%)	10 (16.9%)
**pN**		
pN2	54 (100%)	56 (94.9%)
pN3	0 (0%)	3 (5.1%)
**pM**		
pM0	52 (96.3%)	51 (86.4%)
pM1	2 (3.7%)	8 (13.6%)
**Overall survival, months (median)**	0–60 (18)	0–60 (22)

a LUSC, lung squamous cell carcinoma; LUAD, lung adenocarcinoma; p, prefix indicating pathological TNM staging; T, descriptor of the extension of the primary tumor; N, descriptor for the presence and extent of lymph node metastases; M, descriptor for the presence of distant metastases.

### High Mass-Resolution MALDI-FT-ICR-MSI Analysis

The tissue preparation steps for high mass-resolution MALDI-FT-ICR-MSI were conducted as previously described.^107^ In brief, TMAs were created by cutting FFPE tissue blocks into 4 μm sections (Microm, HM340E; Thermo Fisher Scientific, Waltham, MA, USA) and mounting sections onto indium tin oxide–coated conductive glass slides (Bruker Daltonik GmbH, Bremen, Germany) that were preprocessed with 1:1 poly-l-lysine (Sigma–Aldrich, Munich, Germany) and 0.1% Nonidet P-40 (Sigma–Aldrich). The TMAs were then incubated for 1 h at 60 °C, deparaffinized twice in xylene for 8 min, and allowed to air-dry at room temperature (15–25 °C). Subsequently, a matrix of 10 mg/mL 9-aminoacridine (9-AA) in 70% methanol (purchased from Sigma–Aldrich, Munich, Germany) was sprayed onto the TMA using a SunCollect sprayer (Sunchrom, Friedrichsdorf, Germany) in eight passes using a line distance of 2 mm and a spray velocity of 900 mm/min, with ascending flow rates of 10, 20, and 30 μL/min for passes one to three and a constant flow rate of 40 μL/min for passes four to eight. The detailed MALDI-MSI and matrix application procedures can be found in the protocol by Ly and Buck et al.^107^

MALDI-MSI was performed in negative ion mode on a 7 T Solarix XR FT-ICR mass spectrometer (Bruker Daltonik) equipped with a dual ESI-MALDI source and a SmartBeam-II Nd: YAG (355 nm) laser. The MALDI-MSI data acquisition parameters were specified by the ftmsControl 2.2 and flexImaging (v 5.0) software (Bruker Daltonik, Bremen, Germany). Mass spectra were acquired over a mass range of *m*/*z* 75–1000 Da and at a spatial resolution of 50 μm. Regions without tissues were measured as background controls to differentiate between tissue- and matrix-associated peaks. Exterior calibration of the instrument was performed using l-arginine (Sigma–Aldrich) in the ESI mode. Internal mass calibration was performed by using the 9-AA matrix ion signal (*m*/*z* 193.077122). We used our recently published SPACiAL approach for immunophenotype-guided analysis to identify tumor and stromal compartments and automatically segmented carbon particles into tumor or stromal compartments.^108^ SPACiAL is a computational multimodal workflow that uses a series of image and MALDI data-processing steps to combine molecular imaging data with multiplex immunofluorescence. The SPACiAL workflow includes MALDI and immunofluorescence data integration, multiple image coregistration, image digitization, and data conversion. In brief, after MALDI-MSI analysis, the 9-AA matrix was removed from TMA sections by being soaked in 70% ethanol for 5 min. The TMA sections were then double stained for immunofluorescence analysis with pan-cytokeratin (monoclonal mouse pan-cytokeratin plus [AE1/AE3 + 8/18], 1:75, catalog no. CM162, Biocare Medical, USA) and vimentin (Abcam, clone ab92547, 1:500). Tumor regions were defined by pan-cytokeratin positivity, whereas stromal regions were defined by pan-cytokeratin negativity and vimentin positivity.

### Data Acquisition and Processing

MALDI mass spectra were root mean-square normalized using SCiLS Lab (v 2020b Pro, Bruker Daltonik), and picked peaks were exported as imzML files for subsequent data processing. Peaks were annotated by accurate mass matching with the Human Metabolome Database (HMDB; http://www.hmdb.ca/) and the Kyoto Encyclopedia of Genes and Genomes database (KEGG; https://www.genome.jp/kegg/),^109^ allowing M–H, M–H_2_O–H, M+K–2H, M+Na–2H, and M+Cl as negative adducts with a mass tolerance of 4 ppm. Compounds with indications of being drugs, pesticides, plants, or other irrelevant substances were directly excluded.

### Quantification of Anthracosis Particles

TMA sections were counterstained with nuclear red stain (Fluka, 60700, 0.1%). The stained sections were scanned using an AxioScan.Z1 digital slide scanner (Zeiss, Jena, Germany) equipped with a 20× magnification objective and visualized with the ZEN 2.3 blue edition software (ZEISS, Oberkochen, Germany). Anthracotic particles were quantified by digital image analysis using the Definiens Developer XD2 software (Definiens AG, Germany) as previously described.^110^ The ratio of the particle area to total tissue area was calculated for each tissue core.

### Metabolic Correlation Networks

The relationships among levels of the 11 exogenous compounds and various endogenous metabolites were characterized using pairwise Spearman’s rank–order correlation (Python 3.8, SciPy 1.7.1). The resulting *p* values were adjusted with the Benjamini–Hochberg corrections (Python 3.8, StatsModels 0.13.1) and filtered for significance (*p* < 0.01). The resulting correlation coefficients were visualized as metabolic networks using Cytoscape (v. 3.8.0).^111^

### Statistical Analysis

Statistical analyses were performed using R (https://cran.r-project.org) and Python software (https://www.python.org) with suitable packages. Pairwise Spearman’s rank–order correlations between exogenous molecules and endogenous metabolites were conducted using R 4.0.2, corTest. Significant differences between variables were determined by the rank-based Mann–Whitney U test. Kaplan–Meier analyses and Cox proportional hazards regression analyses were used to determine statistical differences in patient survival, with corresponding *p* values based on the log-rank test (R 4.0.2, survival). “Cutoff-optimized” in this context means that the thresholds for low and high levels of a given compound were chosen such that the *p*-value in the resulting Kaplan–Meier curve was minimal. The profiles of the 11 carbon-bound exogenous compounds were subjected to hierarchical clustering to create a heatmap (R 4.0.2, MetaboAnalystR 3.2). Exogenous compounds were visualized in low-dimensional space using UMAP analysis (Python 3.8, umap).^112^ The significance threshold was set at *p* < 0.05.

## Data Availability

The data sets generated or analyzed during the present study are available at reasonable request from the corresponding authors.

## Supporting Information Available

Figure S1: Carbon-bound compounds in the stromal compartment; Figure S2: Comparison of carbon-bound compounds between normal and tumor tissues; Figure S3: Patient outcomes are associated with the exogenous compounds; Figure S4: Correlations of NNK with the metastasis; Figure S5: Comparison of the number of carbon particles in two tumors; Figure S6: Correlations between NNAL, NNAL-glucuronide, and carbon particles (PDF)
