# Supplementary material for: Patterns of Carbon-Bound Exogenous Compounds Impact Disease Pathophysiology in Lung Cancer Subtypes in Different Ways
Source: ACS Nano. 2023 Aug 28;17(17):16396–411. doi: 10.1021/acsnano.2c11161 (PMC10510585; doi:10.1021/acsnano.2c11161)
Supplement: Supplementary file 1 — nn2c11161_si_001.pdf [file nn2c11161_si_001.pdf]

Supporting Information

Patterns of carbon-bound exogenous compounds impact disease pathophysiology in lung cancer subtypes in different ways

Jian Shen[1,2]†, Na Sun[1]†, Jun Wang[1]†, Philipp Zens[3, 4], Thomas Kunzke[1], Achim Buck[1], Verena M Prade[1], Qian Wang[1], Annette Feuchtinger[1], Ronggui Hu[5], Sabina Berezowska[3, 6], and Axel Walch[1]**

[1] Research Unit Analytical Pathology, Helmholtz Zentrum München– German Research Center for Environmental Health, Neuherberg 85764, Germany

[2] Nanxishan Hospital of Guangxi Zhuang Autonomous Region, Institute of Pathology, Guilin 541002, People's Republic of China

[3] Institute of Tissue Medicine and Pathology, University of Bern, Murtenstrasse 31, Bern 3008, Switzerland

[4] Graduate School for Health Sciences, University of Bern, Mittelstrasse 43, Bern 3012, Switzerland

[5] Center for Excellence in Molecular Cell Science, Chinese Academy of Sciences, Shanghai 200030, People's Republic of China

[6] Department of Laboratory Medicine and Pathology, Institute of Pathology, Lausanne University Hospital and University of Lausanne, Lausanne 1011, Switzerland

0.001.

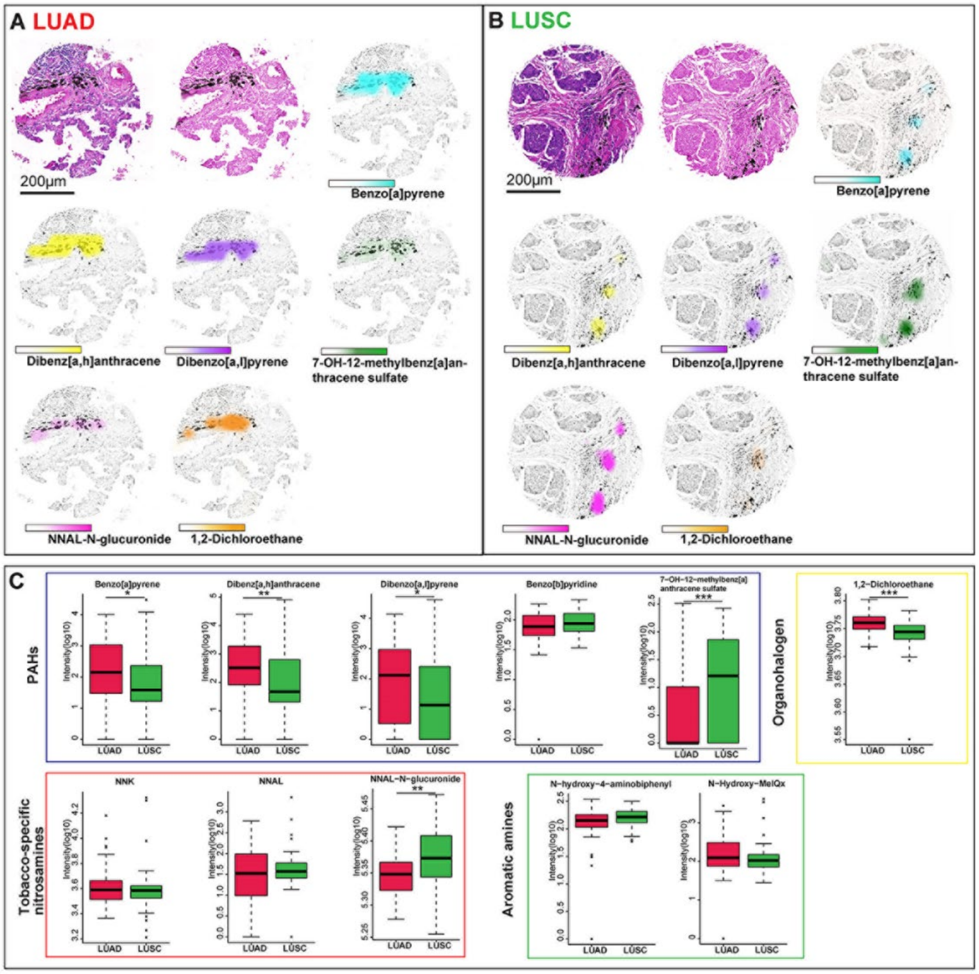

Figure S1. Different patterns of carbon-bound compounds between LUAD and LUSC in the stroma compartment. A and **B**, LUAD and LUSC stroma tissues featuring high carbon-particle content (top left in each panel, hematoxylin and eosin staining and nuclear red staining) and ion distributions of benzo[a]pyrene, dibenz[a,h]anthracene, dibenzo[a,l]pyrene, and 1,2-Dichloroethane were significantly more abundant in LUAD. Conversely, 7-OH-12-methylbenz[a]anthracene sulfate and NNAL-N-glucuronide were more abundant in LUSC. **C**, Comparison of the patterns of exogenous compounds between LUAD and LUSC. LUAD, lung adenocarcinoma; LUSC, lung squamous cell carcinoma; NNAL, 4-(methylnitrosamino)-1-(3-pyridyl)-1-butanol; NNK, nicotine-derived nitrosamine ketone; * $p < 0.05$, ** $p < 0.01$, *** $p < 0.001$.

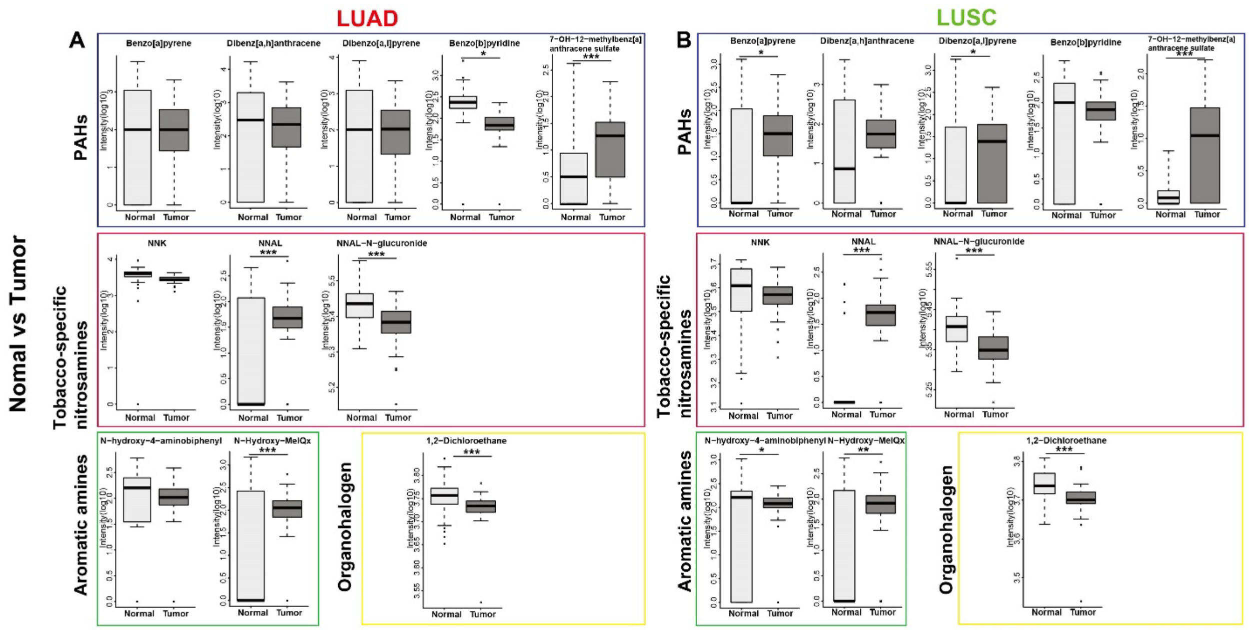

Figure S2. Different patterns of carbon-bound compounds between LUAD and LUSC tumor compartments and normal lung tissue. A, Comparison of quantities of carbon-bound compounds between normal tissues and tumor tissues in LUAD**. B,** Different quantities of carbon-bound compounds within normal tissues and tumor tissues in LUSC. LUAD, lung adenocarcinoma; LUSC, lung squamous cell carcinoma; NNAL, 4-(methylnitrosamino)-1-(3-pyridyl)-1-butanol; NNK, nicotine-derived nitrosamine ketone; * $p < 0.05$, ** $p < 0.01$, *** $p < 0.001$.

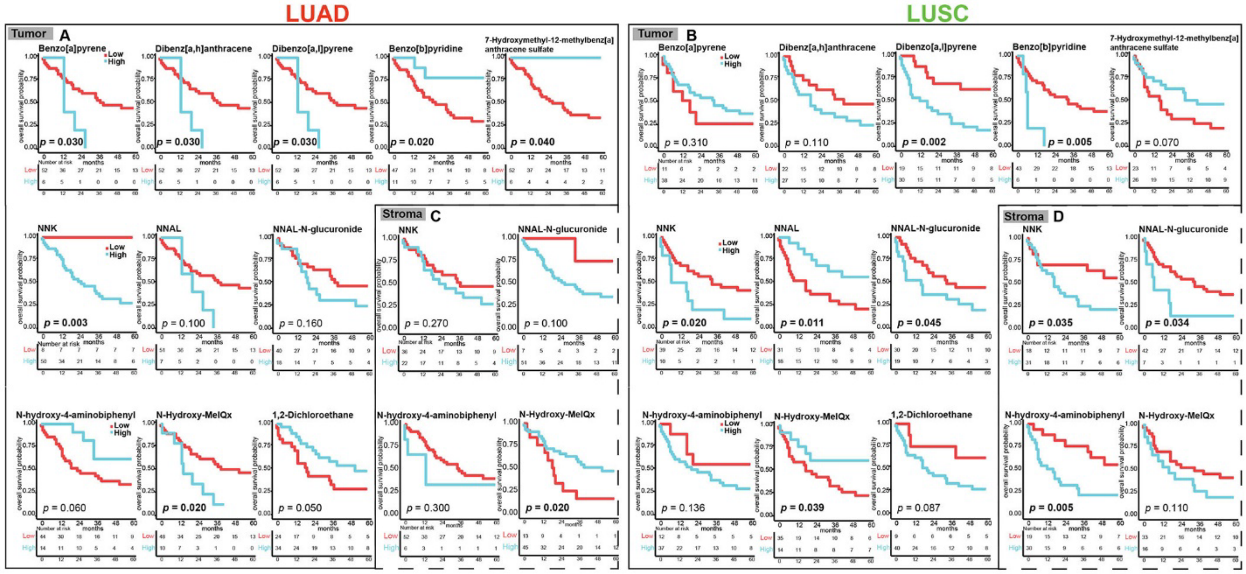

Figure S3. Patient outcomes were differently associated with the amounts of exogenous compounds in LUAD and LUSC. A and B, In Kaplan-Meier survival analyses, exogenous compounds in the tumor epithelial compartment were associated with patient outcomes in LUAD and LUSC**. C and D,** Exogenous compounds in the stroma compartment were associated with patient outcomes in LUAD and LUSC. LUAD, lung adenocarcinoma; LUSC, lung squamous cell carcinoma; NNAL, 4-(methylnitrosamino)-1-(3-pyridyl)-1-butanol; NNK, nicotine-derived nitrosamine ketone.

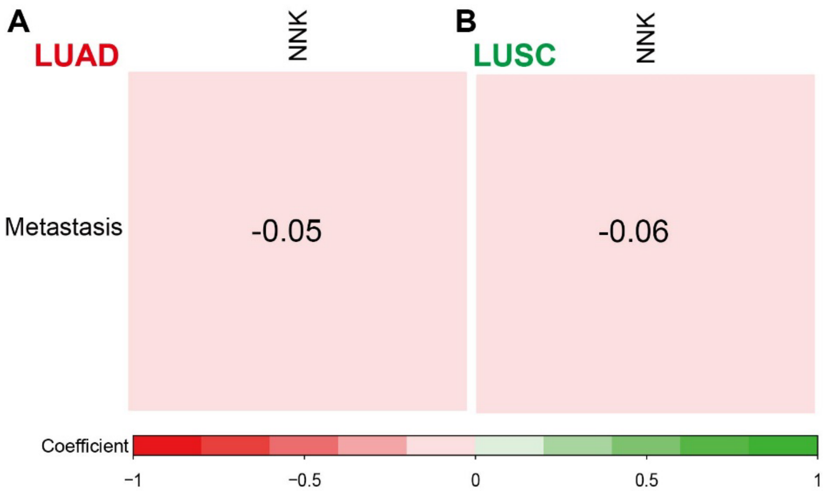

Figure S4. Correlations of NNK with the exogenous compounds in LUAD and LUSC. There is no associated between NNK and metastasis in both LUAD (*p* = 0.96, coefficient = -0.05) **(A)** and LUSC (*p* = 0.83, coefficient = -0.06) **(B).** LUAD, lung adenocarcinoma; LUSC, lung squamous cell carcinoma; NNK, nicotine-derived nitrosamine ketone.

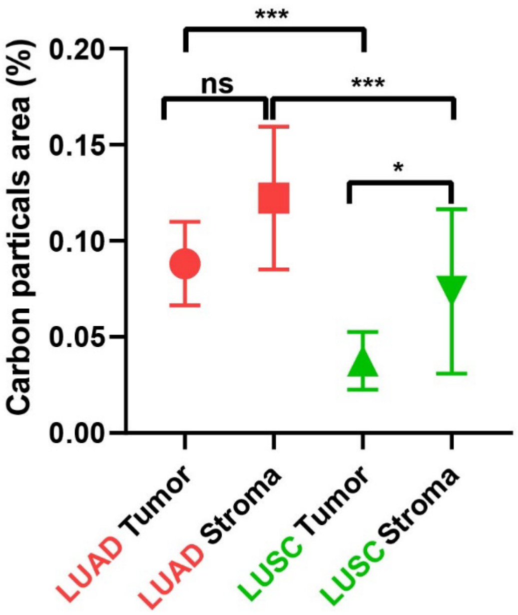

Figure S5. Comparison of the number of carbon particles between LUAD and LUSC. Carbon particles in both tumor and stromal tissue were more abundant in LUAD than in LUSC ($p < 0.001$). In LUAD, there were no significant differences in carbon particles between tumor and stromal tissue, whereas in LUSC, carbon particles were more abundant in the stromal tissue compared to the tumor tissue. LUAD, lung adenocarcinoma; LUSC, lung squamous cell carcinoma; ns, not significant; * $p < 0.05$, ** $p < 0.01$, *** $p < 0.001$.

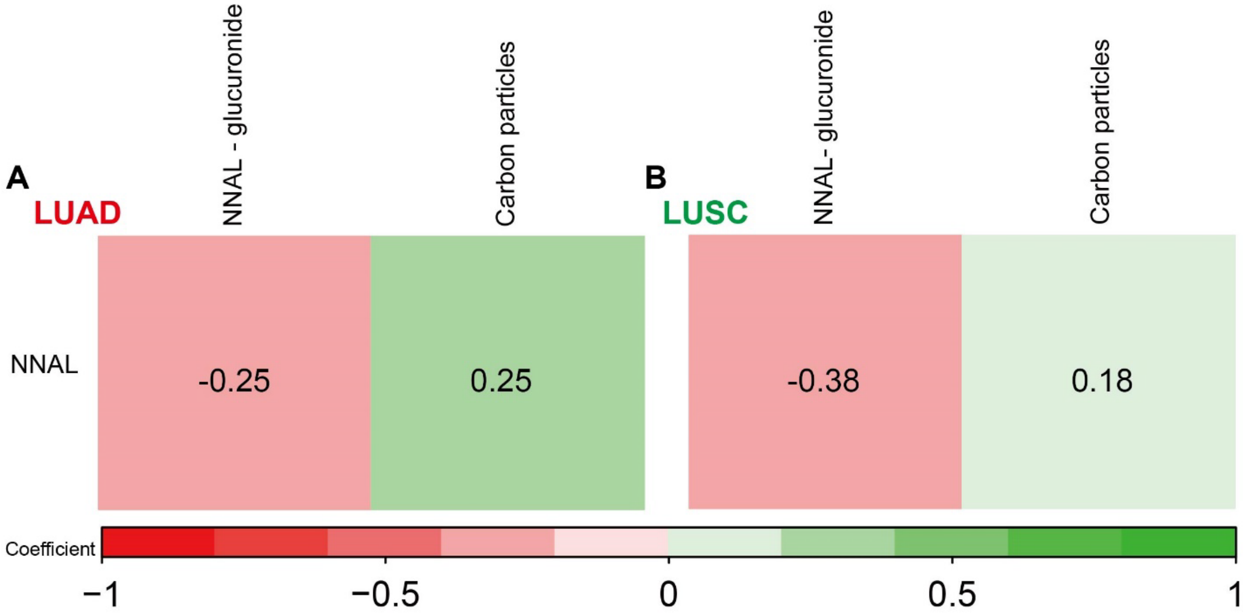

Figure S6. Correlations of NNAL with the NNAL-glucuronide and carbon particles in LUAD and LUSC. There is no associated between NNAL and NNAL-glucuronide in both LUAD (p = 0.14, coefficient = -0.25) **(A)** and LUSC (p = 0.77, coefficient = -0.38) **(B).** There is no associated between NNAL and carbon particles in both LUAD (p = 0.14, coefficient = 0.25) **(A)** and LUSC (p = 0.44, coefficient = 0.18) **(B).** LUAD, lung adenocarcinoma; LUSC, lung squamous cell carcinoma; NNAL, 4-(methylnitrosamino)-1-(3-pyridyl)-1-butanol.
